# Craniofacial Growth at Age 6–11 Years after One-Stage Cleft Lip and Palate Repair: A Retrospective Comparative Study with Historical Controls

**DOI:** 10.3390/children9081228

**Published:** 2022-08-13

**Authors:** Benito K. Benitez, Seraina K. Weibel, Florian S. Halbeisen, Yoriko Lill, Prasad Nalabothu, Ana Tache, Andreas A. Mueller

**Affiliations:** 1 Oral and Craniomaxillofacial Surgery, University Hospital Basel, Spitalstrasse 21, 4031 Basel, Switzerland; 2 Facial and Cranial Anomalies Research Group, Department of Clinical Research, University of Basel, Spitalstrasse 12, 4031 Basel, Switzerland; 3 Facial and Cranial Anomalies Research Group, Department of Biomedical Engineering, Biomaterials Science Centre, University of Basel, Gewerbestrasse 14, 4123 Allschwil, Switzerland; 4 Surgical Outcome Research Center Basel, University Hospital and University of Basel, Spitalstrasse 12, 4031 Basel, Switzerland; 5 Cleft & Craniofacial Team, Universitair Ziekenhuis Brussel, Vrije Universiteit Brussel, 1090 Brussel, Belgium

**Keywords:** cleft lip, cleft palate, growth and development, treatment outcome, cephalometry

## Abstract

Background: Primary alveolar bone grafting inhibits craniofacial growth. However, its effect on craniofacial growth in one-stage cleft lip and palate protocols is unknown. This study investigated whether primary alveolar bone grafting performed during one-stage unilateral cleft lip and palate repair negatively affects growth up to 6–11 years old. Methods: The craniofacial growth, dental arch relationship and palatal morphology at 6–11 years old in children with unilateral cleft lip and palate were compared retrospectively. Two cohorts after a one-stage protocol without (Group A) and with (Group B) primary bone grafting at the same center were compared. Further, cephalometric measurements for growth were compared with an external cohort of a one-stage protocol and a heathy control. Results: Group A comprised 16 patients assessed at 6.8 years (SD 0.83), and Group B comprised 15 patients assessed at 9 years (SD 2.0). Cephalometric measurements indicated similar sagittal maxillary growth deficits and a significant deviation in maxillary inclination in both groups compared to the healthy group. Moderate to severe changes in palatal morphology were observed in 70% of the members in both groups. Conclusion: Omitting primary alveolar bone grafting under the one-stage protocol with two-flap palatoplasty studied did not improve growth at 6–11 years. The results implicate two-flap palatoplasty with secondary healing as having greater adverse effects on growth than primary alveolar bone grafting. Dental and palatal morphology was considerably compromised regardless of primary alveolar bone grafting.

## 1. Introduction

Unoperated adult patients with unilateral cleft lip and palate (UCLP) show a normal craniofacial growth potential at the expense of persistently wide palatal and alveolar clefts [1]. Cleft surgery, especially on the cleft palate, is known for adverse effects on craniofacial growth [2]. To limit growth inhibition by cleft repair, staged protocols have been developed to postpone surgical interventions to time periods with less of an impact on growth [3]. In contrast, one-stage protocols, combining lip and palate closure, focus on reducing patient and parent burden, early normal function, shortening anesthesia time and lowering overall healthcare costs [4,5,6,7]. Technical differences among one-stage protocols might have an influence on craniofacial growth and should therefore be investigated.

Primary alveolar bone grafting, leading to an early connection of the cleft segments, showed negative effects on growth [8,9,10]. Combining primary alveolar bone grafting with primary cleft repair has been controversial [11], and it has been largely abandoned due to its negative effects on growth [8,9]. However, the influence of additional primary alveolar bone grafting in a one-stage cleft lip and palate protocol has not been evaluated.

The study’s purpose and primary objective is to answer the following clinical question: Among patients with UCLP, does primary alveolar rib bone grafting, when compared with no bone grafting at one-stage cleft lip and palate repair, restrict craniofacial growth and affect the dental arch relationship and palatal morphology assessed at 6–11 years.

The secondary objective is to compare craniofacial growth with external historical controls of a one-stage cleft lip and palate surgical protocol and a healthy control group.

The null and alternative hypotheses are as follows:

**H_0_.**  *There is no difference in craniofacial growth assessed at 6–11 years of age between patients after one-stage unilateral cleft lip and palate repair with or without primary alveolar bone grafting.*

**H_a_.**  *There is a significant difference in craniofacial growth assessed at 6–11 years of age between patients after one-stage unilateral cleft lip and palate repair with or without primary alveolar bone grafting.*

## 2. Materials and Methods

### 2.1. Study Design and Setting

A retrospective comparative study between cohorts of children with complete UCLP was performed. The comparison groups were represented by historical controls from the literature. The report follows the STROBE guidelines for observational studies [12]. The setting consisted of two multidisciplinary cleft services in Europe: Group A and Group B—Cleft and Craniofacial Team, University Hospital Basel, Switzerland; Group S—Institute of Mother and Child, Warsaw, Poland.

For Group A and B, the study was approved by the Ethics Commission of Northwest and Central Switzerland (EKNZ) (project-ID 2017-00036 and 2006-00256), and for Group S, the study was approved by the Bioethics Committee at the Institute of Mother and Child, in accordance with the Declaration of Helsinki.

### 2.2. Participants and Procedures

Patients with complete UCLP after one-stage cleft lip and palate repair who had cephalograms from the age of 6–11 years were included in this study. Children with associated syndromes or a lack of consent for the study were excluded. Table 1 shows all the groups compared, along with their treatment protocol and the healthy control. All surgical procedures were performed differently among the groups, but all were performed by experienced single surgeons.

For the primary objective, the craniofacial growth, dental arch relationship and palatal morphology after one-stage cleft lip and palate repair were compared between the groups without (Group A) and with (Group B) primary rib bone grafting.

The treatment protocol of Group A included passive presurgical orthopedic treatment from birth to surgery [16]. One-stage cleft repair was performed at 4–6 months of age, including primary lip repair, cranial pedicled vomer flap and two-flap palatoplasty with secondary healed lateral relaxing incisions. Group A consisted of consecutive patients operated on by the single surgeon A between January 2003 and December 2014.

Group B was previously published; the age-matched subgroup 1 (6–11 years) was included for comparison [13]. The treatment protocol of Group B was identical to that of Group A, except for the surgery at 6 months old and additional primary rib bone grafting [6]. This cohort consisted of consecutive patients operated on by surgeon B between January 1991 to December 2002.

For the secondary objective, the comparison included external historical controls.

Group S was previously published by the Warsaw center in the Slav-cleft study [14]. The treatment protocol of Group S included no presurgical orthopedics. The one-stage surgical closure (lip and palate) at 9 months of age by the same surgeon comprised: lip closure (triangular flap), hard and soft palate repair with bipedicled flaps, medial extended vomer flap, hamulus fracture and nasal mucosa and muscle-aponeurosis detachment from the posterior hard palate [17]. The cohort consisted of children with complete UCLP operated on between 1994 and 1996 by a single experienced surgeon. Lateral cephalograms at the age of 8–13.6 years were reported for Group S.

As a healthy non-cleft control (Group H), cephalometric standards out of the Atlas of Craniofacial Growth from the University School Growth Study were included [15]. Lateral cephalograms of children aged 6–9 years without a history of orthodontic treatment were analyzed.

**Figure 1:**
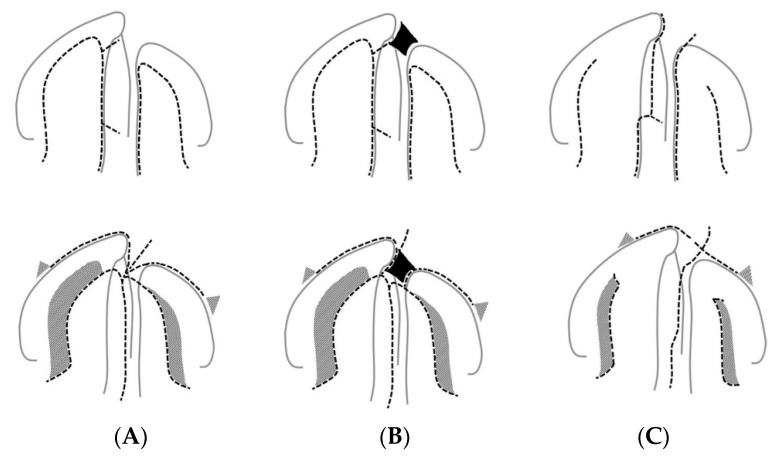
Upper row illustrates incision outlines (dashed line), and lower row illustrates the suture outline for the cleft palate repair of Group A (**A**), Group B (**B**) and Group S (**C**) and the site of secondary healing (gray). In Group B (**B**), primary bone grafting with rib bone (black) is shown in the alveolar cleft.

Figure 1 illustrates the surgical procedures for the cleft palate repair of Groups A, B and S with the incision layout and the course of the sutures with secondary healing sites.

### 2.3. Outcome Variables, Data Sources and Measurements

**Figure 2:**
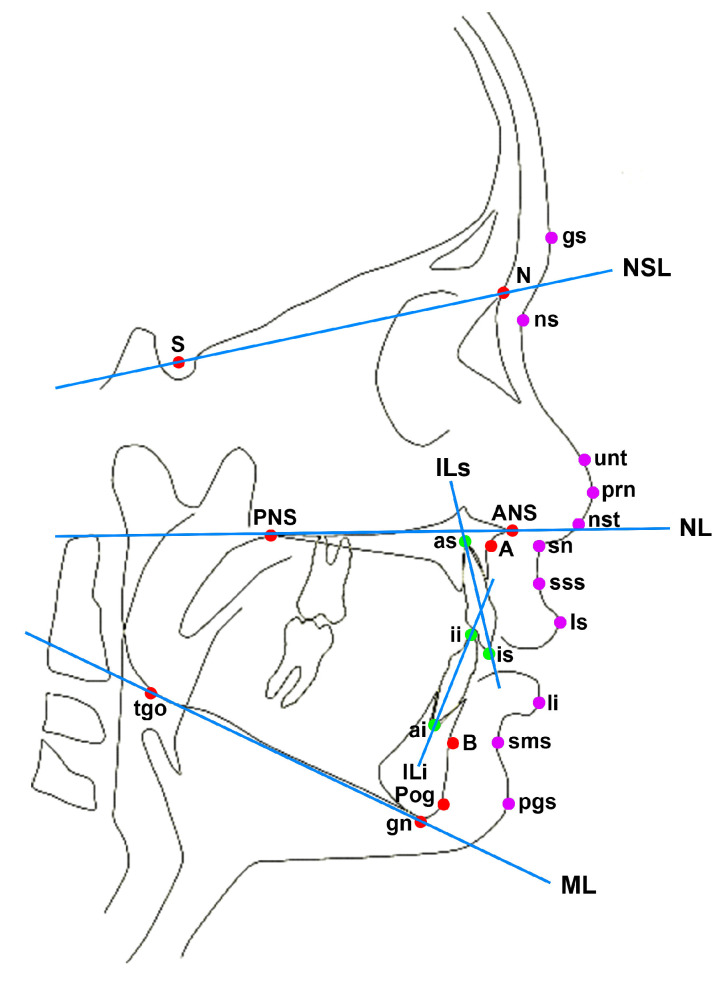
Reference points used for cephalometric analysis: **Skeletal reference points** (red): N—nasion, S—sella, A—subspinale (A-Point), B—supramentale (B-Point), Pog—pogonion, gn—gnathion, tgo—gonion, ANS—spina nasalis anterior, PNS—spina nasalis posterior; **Dental reference points** (green): as—apicale superius, is—inzision superius, ii—inzision inferius, ai—apicale inferius; **Soft tissue reference points** (purple): gs—soft tissue glabella, ns—soft tissue nasion, unt—upper nasal tangent from ns, prn—pronasale, nst—nasal septum tangent point, sn—subnasale, sss—soft tissue subspinale, ls—labrale superius, sms—soft tissue supramentale, pgs—soft tissue pogonion; **Reference lines** (blue): NSL—nasion-sella-line (line through N and S), NL—nasal line (line through PNS and ANS), ML—mandibular line (tangent to the lower border of the mandible trough gn), ILs—axis of upper incisors (line from is to as), ILi—axis of lower incisors (line from ii to ai). Reference points derived from Brattström et al., 2005 [18].

The primary outcome—the craniofacial growth of pre-adolescent children from Groups A, B, S and H—was evaluated based on lateral cephalograms. Figure 2 illustrates the reference points used for cephalometric analysis. To minimize the bias due to different ages, only angular measurements were used. Table A1 in the appendix shows the seven hard tissue and seven soft tissue measurements and their identification in the comparative studies. The lateral cephalograms of Group A were independently assessed by two investigators using OnyxCeph^3TM^ software (Image Instruments, Chemnitz, Germany). This was compared with the previously published results of the lateral cephalometric analysis from Group B with primary rib bone grafting [13], the historical controls of Group S and the healthy control Group H [14,15].

Based on the EUROCRAN Index, we evaluated the dental arch relationship and palatal morphology (EUROCRAN dental and palatal morphology grade) on the dental casts between Groups A and B [19,20]. The absence of the permanent lateral incisor based on photographs, orthopantomography and dental casts was assessed.

### 2.4. Statistical Methods

Descriptive statistics (means, standard deviation) were calculated for Groups A and B. The primary outcome variables of craniofacial growth were analyzed by one-way ANOVA with Tukey Kramer post hoc pairwise tests to identify intergroup differences for angular and ratio variables. Statistical significance was set at *p* < 0.05. The interrater reliability of the cephalometric measurements in Group A by the two evaluators was determined by the interclass correlation coefficient (ICC). An ICC under 0.5 was interpreted as poor, 0.5–0.75 as moderate, 0.75–0.9 as good and >0.90 as excellent reliability [21]. Bland–Altman plots were used for visual representation. Data analysis was performed using STATA 15.0 (StataCorp LLC, College Station, TX, USA) and R statistical software version 3.5.2 (Boston, MA, USA)

## 3. Results

For Group A, forty patients were assessed for eligibility based on medical records. Seven patients were excluded due to missing consent, sixteen patients who lacked a lateral cephalogram at 6–9 years were exluded and one patient was excluded due to a low-quality cephalogram. Therefore, sixteen patients were included and analyzed in Group A.

Table 2 shows the baseline characteristics of Group A (without primary bone grafting) compared to those of Group B (with primary bone grafting), as well as Group S as an external control of a one-stage protocol. Group A was younger at both surgery and assessment (on average, 3.9 months and 6.8 years, respectively) than Group B (6 months and 9 years).

The interrater reliability with ICC for the cephalometric measurements in Group A is shown in Table A2 (Appendix A). The ICC showed a medium to high range (0.57–0.97) of agreement between the two investigators for all variables in Group A. Figure A1 shows the Bland–Altman plots, demonstrating a good agreement between the investigators for the cephalometric variables in Group A, consistent with the findings of the ICC.

### 3.1. Dental Arch Relationship and Palatal Morphology

Table 3 shows the dental arch relationship and palatal morphology for Groups A and B, quantified by the EUROCRAN index and the status of the lateral permanent incisor. Moderate to severe changes in palatal morphology were observed in 70% of members in both groups. In more than 40%, the non-cleft side lateral permanent incisor was missing.

### 3.2. Craniofacial Growth

For the primary objective, the measurements of craniofacial growth from Group A (Cohort 2004–2014) and Group B (Cohort 1991–2002) were compared. The children in both groups exhibited a similar and significant (*p* < 0.001) sagittal growth deficit of the maxilla, with a mean SNA of 76.5° (SD 5.9°) and an SNA of 76° (SD 4°), respectively, compared to the healthy non-cleft control Group H (81° (3.1°)). The maxillary inclination showed a significant difference (*p* < 0.001) from the normal cranial relationship. The angle NSL/NL was larger in both Group A (11.7° (4.2°)) and Group B (14° (4°)) than in Group H (6.4° (2.5°)). The intermaxillary relation ANB was similar between Groups A (3.5°) and B (3°). The only significant difference in the hard tissue between Groups A (88.53° (8.1°)) and B (103° (15°)) was in the inclination of the upper incisor (ILs/NL) (*p* < 0.001). The chin prominence (S-N-Pog) was slightly lower (*p* = 0.77) in Group A (73.9° (4.3°)) than that in Group B (75° (4°)) and Group H (76.1° (2.9°); *p* = 0.03). The nasal profile differed in ns-unt/NSL between Group A (102.4° (7.1°)) and B (107° (4°) (*p* = 0.044)).

Table 4 shows the one-way ANOVA of craniofacial growth in hard and soft tissue among all the groups. Table A3 and Table A4 show the results of the pairwise comparison using the Tukey HD post hoc test.

For the secondary aim, the craniofacial growths of historical and healthy controls were included in the comparisons. A comparable restriction of maxillary growth (SNA) with significantly (*p* < 0.001) smaller SNA in all groups was found compared to the healthy control. The rotation of the upper face (NSL/NL) differed in all groups (*p* < 0.001) from the healthy control. The deviations from the norm were the highest in Group B (Δ = 7.62 (5.66–9.58)), followed by Group A (Δ = 5.3 (3.40–7.20)). The angle measurements related to the mandible were comparable across all groups. ANB was larger (*p* = 0.02) in Group A (3.5° (4.3°)) than in Group S (1.33° (2.8°)), which lagged behind the healthy control (4.8° (2.3°); *p* < 0.001). The interincisal angles (ILs/NL and ILs/Ili) in Group A differed strongly from the others. In the soft tissue morphology, a significantly pronounced facial convexity (gn-sn-pgs) was observed in Group S compared to the other groups (*p* < 0.001).

## 4. Discussion

The study’s purpose was to investigate, among patients with UCLP, whether primary alveolar rib bone grafting (Group B), when compared with no bone grafting (Group A), at one-stage cleft lip and palate repair restricts craniofacial growth assessed at 6–11 years of age. The hypothesis—whether there is no difference in craniofacial growth assessed at 6–11 years of age between patients after one-stage UCLP repair with or without primary alveolar bone grafting—was tested. As a secondary aim, craniofacial growth was compared with the external historical controls of a one-stage cleft lip and palate surgical protocol (Group S) and a healthy control group (Group H).

Our results failed to reject the hypothesis, showing comparable craniofacial growth in Group A (without primary alveolar bone grafting) and Group B (with primary alveolar bone grafting). The comparison between the measurements of the cephalometric radiographs of Groups A and B showed a similar relationship of the maxilla to the skull base, with an indication of craniofacial growth inhibition and alteration from the healthy control.

Eliminating primary alveolar bone grafting in the respective one-stage cleft lip and palate protocol did not improve growth at the time point studied. The present study indicates that the impact of primary alveolar bone grafting itself on craniofacial growth, when performed along with the studied one-stage protocol, is negligible. The only significant difference in hard tissue variables was the inclination of the upper incisors (ILs/NL), explained by the younger age in Group A (6.8 years) prior to the eruption of the permanent incisor compared to that in Group B (9 years) at the time of evaluation.

To answer the question of the influence of the treatment protocol on the dental arch relationship and palatal morphology, the plaster casts of Groups A and B were compared. Likewise, the dental arch relationship and palatal morphology based on the EUROCRAN index were equally altered in Groups A and B. These changes must be attributed to the treatment, as no crowding of the teeth and well-aligned dental arches are reported in unoperated patients with UCLP [22]. Additionally, these results implicate a greater impact on growth by other aspects of the surgical technique compared to the intervention in the alveolar cleft.

An increased number of missing lateral permanent incisors on the non-cleft side in both groups (Group A 56%, Group B 40%) was found. Despite the controversial literature regarding missing teeth outside the cleft [23], the lower prevalence in unoperated adult patients with clefts [24] and the natural prevalence of 3.77% [25] indicate a surgical side effect.

We assessed the craniofacial growth after different one-stage protocols in relation to a healthy group based on external historical data. Our data show a significant restriction of maxillary growth (SNA) and rotation of the upper face (NSL/NL) at 6–11 years old in Groups A, B and S after one-stage surgical protocols.

### 4.1. Clinical Relevance

In summary, these results demonstrate not only sagittal and vertical growth restriction but also the alteration of the transversal growth measured in the dental arch relationship and palatal morphology. As these changes were measured at an age before puberty and the completion of growth, they must be regarded as clinically relevant. Of particular concern is the negative influence of scar formation due to secondary wound healing with the two-flap palatoplasty used in the one-stage protocol in Groups A and B, as depicted in Figure 1. The altered dental arch relationships, as quantified in Groups A and B, might be caused by denuded bony areas in the cleft palate repair [20] influencing subsequent transversal growth, as described in different treatment protocols [26].

Previous studies have reported maxillary retrusion due to primary alveolar bone grafting [8,9,10,11,27,28,29,30], but others have reported successful outcomes when following presurgical orthopedic therapy [27,29,30]. Presurgical therapy with passive plates is known to reduce the cleft of the palate [13]. Nevertheless, two-flap palatoplasty in the subsequent procedure leads to secondary healing. From a clinical point of view, it needs to be further investigated whether presurgical therapy combined with the incision design used in Group S, allocating parts of the vomerine tissue for oral layer repair, can reduce secondary healing.

Thus, the presented study of Groups A and B prompted changes in the surgical protocol at the study center. Primary alveolar bone grafting [10] and one-stage two-flap palatoplasty with secondary healing of lateral releasing incisions were abandoned. Following passive presurgical therapy, a one-stage protocol with bipedicled palatal flaps was implemented and modified for a continuous two-layer closure and primary healing [7].

Although single-stage lip and cleft palate closure protocols showed a similar growth to multistage surgery [31,32], with the advantage of a reduced treatment burden, further investigation on protocols to reduce the negative effect of cleft surgery on maxillary growth and palatal morphology is warranted [33,34]. In summary, the current findings indicate a greater influence of other aspects of the surgical protocol on growth than the intervention in the alveolar cleft. These results should be considered in the further refinement of one-stage cleft lip and palate strategies to avoid negative effects on craniofacial growth and the dental arch relationship. Henceforth, growth outcomes must be complemented by an assessment of speech and hearing as well as the overall treatment burden [32,35].

### 4.2. Limitations

The limitations are the retrospective nature and the small sample size. However, the historical control at a single center before the change in surgical protocol and the external historical control with independent sample were evaluated to strengthen the validity and included the comparison with a healthy control. The similar mandibular growth among independent samples validates our comparison. The surgical dexterity of three different surgeons may override the effects of the surgical technique on craniofacial growth. However, the different cohorts were operated on by the respective experienced surgeons. The unfavorable developmental trend in craniofacial growth was measured at 6–11 years of age and could increase after puberty and later [36]. Speech development and hearing development were not investigated in our study, as Groups A and B used the same hard and soft palate closure technique.

## 5. Conclusions

Omitting primary alveolar bone grafting in the one-stage cleft lip and palate protocol analyzed did not improve growth at 6–11 years. Dental and palatal morphology was considerably compromised regardless of primary alveolar bone grafting.

## Figures and Tables

**Table 1:** Comparison groups with a summary of the treatment protocols and the healthy non-cleft control group.

Study Population (Publication)	Description	*n*	Age Range [Years]	Treatment Protocol
2003–2014	Group A	16	6–9	One-stage cleft repair: lip, vomer flap and two-flap palatoplasty at 6 months
1991–2002 (Group 1) [13]	Group B	15	6–11	One-stage cleft repair: lip, vomer flap and two-flap palatoplasty with primary rib bone grafting at 6 months
Slav-Cleft (Warsaw) [14]	Group S	35	8–13.6	One-stage cleft repair: lip, vomer flap and bipedicled hard and soft palate repair at 9 months
Healthy control group [15]	Group H	83	6–9	N/A

**Table 2:** Baseline characteristics after one-stage repair of unilateral cleft lip and palate without primary bone grafting (Group A) in comparison to (Group B) that with primary bone grafting and Group S.

	Group A (2003–2014) *n* (%)	Group B (1991–2002) *n* (%)	Group S (1994–1996) *n* (%)
Total patients per group	16	15	35
Female	5 (31.25%)	4 (26.67%)	10 (28.57%)
Male	11 (68.75%)	11 (73.33%)	25 (71.43%)
Cleft			
Left	11 (68.75%)	9 (60%)	N/A
Right	5 (31.25%)	6 (40%)	N/A
Age at study (years) [mean (SD)]	6.8 (0.83)	9 (2)	10.6 (1.2)
Age at cleft repair (months) [mean (SD)]	3.9 (0.62)	6 (1)	9

Data for Group B are derived from Mueller et al., 2012 [13], and data from Group S are derived from Urbanova et al., 2016 [14].

**Table 3:** EUROCRAN Index and status of lateral permanent incisors after one-stage repair of unilateral cleft lip and palate without primary bone grafting (Group A) in comparison to (Group B) that with primary bone grafting.

	Group A (2003–2014) *n* = 16	Group B (1991–2002) *n* = 15
EUROCRAN dental grade ^a^		
1	2 (12.5%)	3 (20%)
2	2 (12.5%)	5 (33%)
3	5 (31.25%)	5 (33%)
4a	6 (37.5%)	2 (13%)
4b	1 (6.25%)	
Mean (SD)	3 (1.0)	2.4 (1.0)
EUROCRAN palatal morphology grade ^b^		
1	5 (31.25%)	3 (20%)
2	8 (50%)	7 (47%)
3	3 (18.75%)	5 (33%)
Mean (SD)	1.9 (0.7)	2.1 (0.7)
Missing lateral incisors—Cleft side [*n* (%)]		
Yes	8 (50%)	11 (73%)
No	8 (50%)	4 (27%)
Missing lateral incisors—Non-cleft side [*n* (%)]		
Yes	9 (56.25%)	6 (40%)
No	7 (43.75%)	9 (60%)
Missing lateral incisors—Bilateral [*n* (%)]		
Yes	6 (37.5%)	5 (33%)
No	10 (62.5%)	10 (67%)

^a^ EUROCRAN index of dental arch relationship. Grade 1: Apical base relationship—skeletal Class I or Class II. Both central incisors have a positive overjet and overbite, or there is a considerably increased overjet with no overbite (note: it is grade 2 if there are obvious dental compensations). Grade 2: apical base relationship is class I. Non-cleft incisor is in a positive overjet and overbite. Tilting or derotation of the cleft-side incisor would achieve a stable overjet and overbite (note: it is grade 3 if there is a moderate open bite). Grade 3: apical base relationship is edge-to-edge or mild skeletal class III. One or both central incisors are edge-to-edge or in a close anterior cross-bite. Tilting or derotation would not achieve a stable overjet and overbite (note: it is grade 4 if there is a severe open bite or if the edge-to-edge position of the incisor in class III is achieved by dental compensation). Grade 4a: apical base relationship is class III. Both central incisors are in an anterior crossbite, or one is in an anterior crossbite with the other being edge-to-edge. Grade 4b: same as grade 3 but with a marked open bite. ^b^ EUROCRAN index of palatal morphology. Grade 1: Good anterior and posterior height; minor surface irregularities (bumps, crevices); nil or minor deviation of the arch form. Grade 2: Moderate anterior and posterior height; moderate surface irregularities (bumps, crevices); moderate deviation of the arch form (e.g., segmental displacement). Grade 3: Severe reduction in palate height; severe surface irregularities (bumps, crevices); severe deviation in the arch form (e.g., “hourglass” constriction). Data for Group B are derived from Mueller et al., 2012 [13].

**Table 4:** Cephalometric values of Group A without primary bone grafting and Group B with primary bone grafting compared with the mean values of the Slav-cleft study (Warsaw center) and the healthy cephalometric standard control values. Data are presented as the mean (SD). Angles are measured in degrees.

		Group A (2003–2014)	Group B (1991–2002)	Group S (Slav-Cleft)	Healthy Control		
		(*n* = 16)	Group 1 (*n* = 15)	Warsaw (*n* = 35)	(*n* = 83)		
		Mean (SD)	Mean (SD)	Mean (SD)	Mean (SD)	*p*-Value #	Differences *
**Hard tissue**							
maxilla	S-N-A	76.5 (5.9)	76 (4)	75.7 (3.6)	81 (3.1)	<0.001	A-H, B-H, S-H
	NSL/NL	11.7 (4.2)	14 (4)	11.2 (4.3)	6.4 (2.5)	<0.001	A-H, B-S, B-H, S-H
mandible	S-N-Pog	73.9 (4.3)	75 (4)	75.4 (4)	76.1 (2.9)	0.022	A-H
	NSL/ML	34.9 (5.5)	35 (4)	37.3 (5.6)	35.1 (4.6)	0.076	-
maxillomandibular	A-N-B	3.5 (4.3)	3 (3)	1.3 (2.8)	4.8 (2.3)	<0.001	A-S, B-H S-H
	ILs/NL	88.5 (8.1)	103 (15)	105 (8.2)	107.3 (7.6)	<0.001	A-B, A-S, A-H
	ILs/ILi	161.1 (11.4)	154 (12)	143 (10.9)	131.7 (11.8)	<0.001	A-S, A-H, B-S, B-H, S-H
**Soft tissue**							
maxillomandibular	sss-ns-sms	5.3 (4.1)	6 (3)	5.9 (2.7)	-	0.79	-
	sss-ns-pgs	4.8 (3.5)	5 (3)	4.5 (3.1)	-	0.86	-
	gs-sn-pgs	187.9 (9.5)	187 (7)	173.5 (6.8)	-	<0.001	A-S, B-S
nasal profile	gs-prn-pgs	149.7 (7.4)	150 (5)	147.8 (5.8)	-	0.38	-
	ns-unt/NSL	102.4 (7.1)	107 (4)	105.9 (4.7)	-	0.036	A-B
	ns-prn-sn	107.5 (4.7)	105 (6)	104.5 (5.9)	-	0.22	-
	nst-sn-ls	107.8 (14.8)	102 (11)	101.6 (12.8)	-	0.27	-

# One-way analysis of variance (ANOVA) analysis. * Tukey’s HSD post hoc test, showing differences between Groups A, B, S and healty control (H). Data for Group B are derived from Mueller et al., 2012 [13], data for Group S are derived from Urbanova et al., 2016 [14] and data for the healthy control are derived from Riolo et al., 1979 [15].

**Figure A1:**
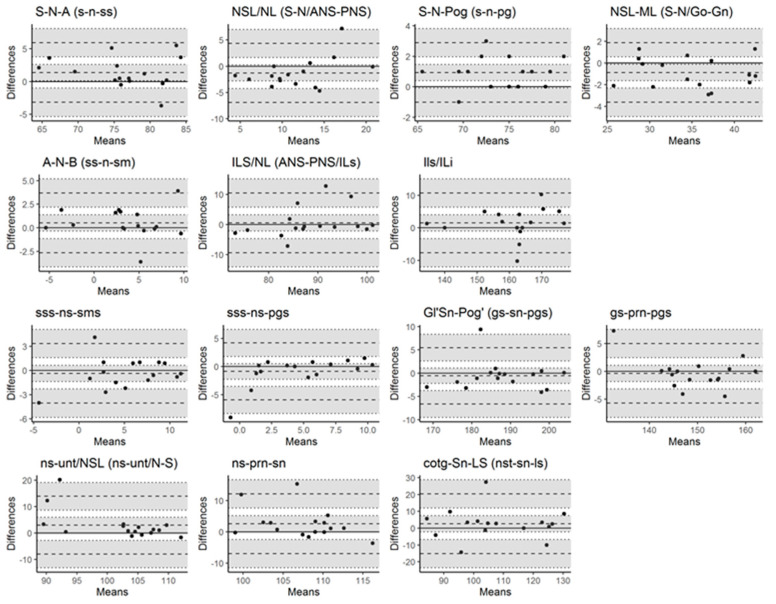
BlandAltman plots demonstrating agreement between the investigators for the cephalometric variables in Group A.

## Data Availability

Not applicable.

## Appendix A

**Table A1:** Variables measured in lateral cephalometric analysis and their identification in comparative studies.

	Group A (2003–2014)	Group B (1991–2002)	Group S (Slav-Cleft)	Healthy Control
**Hard tissue**				
maxilla	S-N-A	S-N-A	s-n-ss (SNA)	A-N-S
	NSL/NL	S-N/ANS-PNS	NSL/NL	N-S/ANS-PNS
mandible	S-N-Pog	S-N-Pog	s-n-pg	PG-N-S
	NSL/ML	S-N/Go-Gn	NSL/ML	N-S/GN-GO
maxillomandibular	A-N-B	A-N-B	ss-n-sm (ANB)	A-N-B
	ILs/NL	ANS-PNS/ILs	Ils/NL	UIE-UIA/PNS-ANS
	ILs/ILi	ILs/ILi	Ils/Ili	LIA -LIE/UIA -UIE
**Soft tissue**				
maxillomandibular	sss-ns-sms	sss-ns-sms	sss-ns-sms	n/a
	sss-ns-pgs	sss-ns-pgs	sss-ns-pgs	n/a
	gs-sn-pgs	gs-sn-pgs	gs-sn-pgs	n/a
nasal profile	gs-prn-pgs	gs-prn-pgs	gs-prn-pgs	n/a
	ns-unt/NSL	ns-unt/N-S	ns-unt/NSL	n/a
	ns-prn-sn	ns-prn-sn	ns-prn-sn	n/a
	nst-sn-ls	nst-sn-ls	nst-sn-ls	n/a

**Table A2:** Interclass correlation between the investigators in Group A.

		ICC (95% CI)
**Hard tissue**		
maxilla	S-N-A	0.92 (0.8–0.97)
	NSL/NL	0.79 (0.5–0.92)
mandible	S-N-Pog	0.97 (0.92–0.99)
	NSL/ML	0.97 (0.91–0.99)
maxillomandibular	A-N-B	0.93 (0.81–0.98)
	ILs/NL	0.82 (0.56–0.93)
	ILs/ILi	0.92 (0.78–0.97)
**Soft tissue**		
maxillomandibular	sss-ns-sms	0.9 (0.74–0.96)
	sss-ns-pgs	0.76 (0.44–0.91)
	gs-sn-pgs	0.95 (0.86–0.98)
nasal profile	gs-prn-pgs	0.93 (0.82–0.98)
	ns-unt/NSL	0.73 (0.39–0.9)
	ns-prn-sn	0.57 (0.13–0.83)
	nst-sn-ls	0.83 (0.58–0.94)

Intraclass correlations (ICC) for Group A between the two investigators show a medium to high range of agreement for all variables.

**Table A3:** Pairwise comparisons of hard tissue variables between the groups using the Tukey HD post hoc test *.

	*n*	Mean	*n*	Mean	Difference	*p*-Value
**S-N-A**						
Group A vs. Group B	16	76.49	15	76	0.49 (−2.62–3.60)	0.98
Group A vs. Group S	16	76.49	35	75.66	0.83 (−1.79–3.45)	0.85
Group A vs. Healthy control	16	76.49	295	81.05	−4.55 (−6.78–−2.33)	<0.001
Group B vs. Group S	15	76	35	75.66	0.34 (−2.34–3.02)	0.99
Group B vs. Healthy control	15	76	295	81.05	−5.05 (−7.34–−2.75)	<0.001
Group S vs. Healthy control	35	75.66	295	81.05	−5.39 (−6.94–−3.83)	<0.001
**NSL/NL**						
Group A vs. Group B	16	11.68	15	14	−2.32 (−4.99–0.34)	0.11
Group A vs. Group S	16	11.68	35	11.24	0.44 (−1.80–2.68)	0.96
Group A vs. Healthy control	16	11.68	294	6.38	5.3 (3.40–7.20)	<0.001
Group B vs. Group S	15	14	35	11.24	2.76 (0.47–5.05)	0.01
Group B vs. Healthy control	15	14	294	6.38	7.62 (5.66–9.58)	<0.001
Group S vs. Healthy control	35	11.24	294	6.38	4.86 (3.54–6.19)	<0.001
**S-N-Pog**						
Group A vs. Group B	16	73.91	15	75	−1.09 (−4.01–1.82)	0.77
Group A vs. Group S	16	73.91	35	75.41	−1.5 (−3.95–0.94)	0.39
Group A vs. Healthy control	16	73.91	294	76.1	−2.2 (−4.28–0.12)	0.03
Group B vs. Group S	15	75	35	75.41	−0.41 (−2.91–2.09)	0.97
Group B vs. Healthy control	15	75	294	76.1	−1.1 (−3.25–1.04)	0.55
Group S vs. Healthy control	35	75.41	294	76.1	−0.69 (−2.14–0.76)	0.61
**A-N-B**						
Group A vs. Group B	16	3.53	15	3	0.53 (−1.78–2.84)	0.94
Group A vs. Group S	16	3.53	35	1.33	2.2 (0.26–4.14)	0.02
Group A vs. Healthy control	16	3.53	294	4.78	−1.25 (−2.90–0.40)	0.21
Group B vs. Group S	15	3	35	1.33	1.67 (−0.31–3.65)	0.13
Group B vs. Healthy control	15	3	294	4.78	−1.78 (−3.48–0.08)	0.04
Group S vs. Healthy control	35	1.33	294	4.78	−3.45 (−4.60–−2.30)	<0.001
**ILs/NL**						
Group A vs. Group B	16	88.53	15	103	−14.47 (−21.96–−6.97)	<0.001
Group A vs. Group S	16	88.53	35	105.02	−16.49 (−22.78–−10.20)	<0.001
Group A vs. Healthy control	16	88.53	294	107.25	−18.72 (−24.07–−13.37)	<0.001
Group B vs. Group S	15	103	35	105.02	−2.02 (−8.46–4.42)	0.85
Group B vs. Healthy control	15	103	294	107.25	−4.25 (−9.77–1.27)	0.19
Group S vs. Healthy control	35	105.02	294	107.25	−2.23 (−5.67–1.50)	0.41
**ILs/ILi**						
Group A vs. Group B	16	161.15	15	154	7.15 (−3.73–18.02)	0.33
Group A vs. Group S	16	161.15	35	143.03	18.12 (8.98–27.25)	<0.001
Group A vs. Healthy control	16	161.15	293	131.75	29.4 (21.63–37.17)	<0.001
Group B vs. Group S	15	154	35	143.03	10.97 (1.63–20.31)	0.01
Group B vs. Healthy control	15	154	293	131.75	22.25 (14.24–30.27)	<0.001
Group S vs. Healthy control	35	143.03	293	131.75	11.28 (5.87–16.70)	<0.001

* Only for angles with a statistically significant difference in the ANOVA analysis.

**Table A4:** Pairwise comparisons of soft tissue variables between the groups using the Tukey HD post hoc test *.

	*n*	Mean	*n*	Mean	Difference	*p*-Value
**gs-sn-pgs**						
Group A vs. Group B	16	187.87	15	187	0.87 (−5.57–7.40)	0.95
Group A vs. Group S	16	187.87	35	173.54	14.33 (8.84–19.81)	<0.001
Group B vs. Group S	15	187	35	173.54	13.46 (7.85–19.07)	<0.001
**ns-unt/NSL**						
Group A vs. Group B	16	102.38	15	107	−4.63 (−9.15–0.10)	0.044
Group A vs. Group S	16	102.38	35	105.91	−3.54 (−7.33–0.26)	0.07
Group B vs. Group S	15	107	35	105.91	1.09 (−2.79–4.97)	0.78

* Only for angles with a statistically significant difference in the ANOVA analysis.
